# ENSO, Nest Predation Risk, Food Abundance, and Male Status Fail to Explain Annual Variations in the Apparent Survival Rate of a Migratory Songbird

**DOI:** 10.1371/journal.pone.0113844

**Published:** 2014-11-24

**Authors:** Alizée Vernouillet, Marc-André Villard, Samuel Haché

**Affiliations:** 1 Département de biologie, Université de Moncton, Moncton, Canada; 2 Department of Biological Sciences, University of Alberta, Edmonton, Canada; University of Lleida, Spain

## Abstract

Adult mortality can be a major driver of population decline in species whose productivity is relatively low. Yet, little is known about the factors influencing adult survival rates in migratory bird species, nor do we know much about the longer-term effects of habitat disturbance on the fitness of individuals. The Ovenbird (*Seiurus aurocapilla*) is one of the vertebrate species most sensitive to forest management, yet it is still common and widespread. We monitored the fate of 330 colour-banded Ovenbird males in four pairs of 25-ha plots during 9 successive breeding seasons. One plot of each pair was treated through selection harvesting (30–40% basal area removed) during the first winter. We tested the following hypotheses: (1) higher physiological costs in harvested plots as a result of lower food abundance will reduce apparent survival rate (ASR) relative to controls; (2) lower ASR following years with low nest survival and higher probability of renesting; (3) fluctuations in ASR reflecting El Niño Southern Oscillation (ENSO); and (4) higher ASR in returning males than in recruits (unbanded immigrants) owing to greater site familiarity in the former. We tested the relative importance of these hypotheses, or combinations thereof, by generating 23 models explaining variation in ASR. The year-dependent model received the most support, showing a 41% decrease in ASR from 2007 to 2014. The important year-to-year variation we observed in ASR (Σ*w_i_* = 0.99) was not explained by variation in nest predation risk nor by ENSO. There was also little evidence for an effect of selection harvesting on ASR of Ovenbird males, despite a slight reduction in lifespan relative to males from control plots (2.7 vs 2.9 years). An avenue worth exploring to explain this intriguing pattern would be to determine whether conditions at migratory stopover sites or in the wintering area of our focal population have gradually worsened over the past decade.

## Introduction

As the human footprint expands across the world, it is important to understand the mechanisms underlying species responses to mitigate negative effects on ecological integrity. Along with immigration and emigration, adult survival influences population growth rates and dynamics [1]. Knowing the relative importance of those parameters is important when developing conservation targets at the species level [2]. Identifying the causes underlying population trends is especially important when considering the dramatic declines observed in several bird species over the past decades [3]–[4].

According to the Sherry-Holmes model [5], the main extrinsic factors influencing survival rate of adult songbirds during the breeding season are high rates of predation (see also [6]), food abundance, and climate. Those three factors influence habitat quality, which in turn can influence the physiological condition of individuals [7] and, thus, their fitness [8]. Effects of various habitat alterations on habitat quality have mainly been documented through short-term (ca. 3 years) monitoring following experimental treatments. Hence, the ultimate effects of habitat disturbance on populations of long-lived species are essentially unknown. Relatively few long-term studies have been conducted owing to the extensive resighting effort needed and the difficulty of securing such data on marked individuals while accounting for emigration ([9]–[10], but see [11]–[12]). Multistate and robust design models have been used to address this challenge (e.g. [13]).

Apparent survival rate (ASR) is generally defined as the local survival rate, i.e. the probability that individuals will survive and return to the area where they were captured [14] and, therefore, it represents a minimum estimate of the actual survival rate because it does not account for permanent emigration [1]. Individuals are more likely to emigrate following an unsuccessful breeding attempt [15]–[17], which in turn would decrease ASR. Because nest predation is the main cause of nesting failure [18], breeding seasons characterized by low nesting success should be followed by a lower ASR than more successful breeding seasons. Males may also suffer a cost when they have to feed multiple broods during the same season following failures at the nestling or early postfledging stages. Alternately, perceived nest predation risk in a given season could promote dispersal [19]. ASR can also be influenced by previous experience with a site, where experienced individuals, i.e. those returning to a site for at least a second breeding season, tend to have a higher return rate than recruits the following year (e.g. [20]) irrespective of breeding success or predation risk, possibly as a result of greater site familiarity (*sensu* [21]).

Global climate cycles, especially the El Niño-Southern Oscillation (ENSO), have also been shown to influence ASR in migratory birds, by changing precipitation patterns and affecting food abundance on the wintering grounds of the Pacific Coast and the Caribbean [22]–[24]. For example, La Niña phases of the ENSO cycle were correlated with adult survival rate in the Yellow Warbler (*Setophaga petechia*) [23], whereas warm and drier conditions during El Niño phases result in food abundance [22] in the Pacific Northwest, the Caribbean and southern Central America [25]. Moreover, in Jamaica, lower rainfall seemed to have a negative impact on overwinter condition of Ovenbirds (*Seiurus aurocapilla*) through their food supply [26]–[27], as well as ASR of Black-throated Blue Warblers (*Setophaga caerulescens*) [28]. Hence, global climatic cycles may influence the return rate of breeding individuals.

Studies documenting ASR in songbirds within a forestry context have mainly focused on short-term effects of landscape context [29]–[32]. These studies report negative effects of habitat loss/fragmentation on ASR, even though habitat within fragments was left intact. In this study, we analyzed the long-term response of an Ovenbird population to an alteration of its breeding habitat by extending the time series of the field experiment undertaken by Haché & Villard [33]. The Ovenbird is a relatively common neotropical migrant songbird that nests on the ground and mainly forages on litter invertebrates in deciduous and mixed-wood forests [34]. It is associated with thick leaf litter and an open understory [35]–[36] and responds negatively to disturbances opening the forest canopy [37]–[38]. The Ovenbird normally produces a single-brood per year, but it will renest after a nest failure occurring early in the season [34]. It mainly winters in Central America and the Caribbean [34]. Haché & Villard [32] monitored the response of individually-marked Ovenbirds before and after their habitat was altered by selection harvesting (30–40% basal area removal). No treatment effect on ASR was detected over a four-year period. However, the density of breeding males in treated plots decreased by 41% in the first year post-harvest owing to a lower recruitment rate. There was a corresponding increase in territory size, which coincided with a decline in the abundance of litter invertebrates [39]. Nonetheless, the harvest treatment had no effect on daily nest survival rate nor on per capita productivity [39]. Although it appears that breeding males from treated plots could be as productive on a per capita basis by foraging over a larger area, this may in turn incur a cost on individuals, possibly reducing their longevity.

This study aimed to quantify the relative importance of factors known to explain inter-annual variation in ASR. We hypothesized that (1) ASR would be lower among males defending a territory in treated plots, owing to the long-term physiological costs associated with lower habitat quality. Within treated plots, we hypothesized that (2) male status (returning from previous year(s) versus recruit) would influence the probability of return owing to greater site familiarity in the former. Hence, we predicted a stronger negative treatment effect on recruits (i.e. significant treatment×status interaction). We further hypothesized that (3) ASR would be lower for all males monitored between years coinciding with low nesting success and the following breeding season, and that (4) fluctuations in ASR would match the ENSO climate pattern over and beyond putative treatment effects. To test these hypotheses, we monitored the fate of the first seven cohorts (i.e. groups of newly-marked males during a breeding season, irrespective of their age) of males banded over a 9-year period.

## Methods

### Study area

This study was conducted in the Black Brook District (47°23′N, 67°40′W), a private managed forest located in northwestern New Brunswick, Canada. We used a before-after control-impact paired (BACIP) study design established in 2006 to quantify the demographic response of songbirds to harvesting. In this study, we surveyed Ovenbirds in 4 pairs of 25 ha study plots from 2006 to 2014. The average distance was 4.2 ±1.0 km (mean ± SD) between paired plots and 23.8±9.1 km among pairs. The study plots were dominated by shade-tolerant deciduous species (sugar maple, *Acer saccharum*; American beech, *Fagus grandifolia*; and yellow birch, *Betula alleghaniensis*). During the winter 2006–2007, one plot of each pair was randomly treated through selection harvesting (30–40% basal area removal - see [33] for details).

### Bird surveys

During seven consecutive breeding seasons (2006–2012), we mist-netted and individually-marked 90% of Ovenbird males. Each plot was intensively surveyed (100 to 200 observer-hours/year) by trained observers (4–6 person crews per year; 5 observers participating to>3 field seasons) from early May to the end of July each year. The fate (returning or not) of each individual was determined by searching both systematically (spot mapping early in the season) and opportunistically (confirming the identify of all males resighted) for unique combinations of colour bands, until all territorial individuals were identified in each plot. We also searched for banded individuals within a ca. 100 m-wide band around each site to find individuals that might have dispersed over a short distance. Each male was also banded with a numbered aluminium band from the Canadian Wildlife Service. We used the approach suggested by Donovan & Stanley ([40], modified by [41]) to age each individual as second-year (SY) or after second-year (ASY) based on the wear pattern of the third rectrix. Females were excluded from this study owing to the difficulty of capturing them. We also excluded males banded in 2013 and 2014 owing to low sample size (i.e. few males were banded). All SY males and unbanded males captured in our sites since 2007 were considered as “recruits” whereas banded individuals returning to the study area are hereafter referred to as “returning males”. Thus, from the first year post-harvest, each male was classified as returning or recruit. Because we could not determine the status of ASY males from the 2006 cohort, we assumed that they were all returning individuals.

### Nesting success

From early May until late July, we searched intensively for nests. All nests were monitored every 3 days to determine their fate (successful, failed or abandoned). A nest was considered successful when fledglings unable to sustain flight were seen nearby or nestlings were at least 8 days old on the penultimate visit and the nest was later found empty and undisturbed. The presence of fecal sacs in the vicinity of the nest was also used to confirm nesting success. A nest was considered depredated if it was found empty before nestlings were old enough to fledge [34]. We estimated annual nesting success and daily nest survival rate using the logistic-exposure method [42] based on the nests found and monitored during a specific breeding season. Estimated values of nesting success were then used for all individuals during a given year. In this study, daily nest survival rate was used as a proxy for nest predation risk and applied to all individuals during a given year.

The banding and nest searching/monitoring protocols were approved by Université de Moncton's Animal Care Committee (12-02) and Canadian Wildlife Service (banding permit 10651 and scientific permit SC2751 to M.-A.V.).

### Climatic influence

To investigate potential effects of climate patterns on annual variations in ASR, we considered El Niño Southern Oscillation (ENSO), more specifically the Southern Oscillation Index (SOI). This index indicates fluctuations in air pressure, and reflects the development and intensity of El Niño. Negative values of SOI are associated with the warm phases of El Niño, whereas positive values correspond to colder, La Niña episodes [25]. SOI values were obtained from the National Oceanic and Atmospheric Administration [43]. We used average SOI values from July to April of for each time interval (e.g. 2006–2007, 2007–2008, etc.) to capture the peak of ENSO, which occurs between October and December [27], to determine its effect on overwinter survival. We did not investigate the effects of ENSO on the breeding grounds because they have been shown to have little influence on the climate of northeastern North America at that time of year [44].

### Statistical analysis

We formulated Cormack-Jolly-Seber models to test whether treatment (t), years (y), status (s), nesting success (ns) and daily nest survival rate (dns), ENSO (e), and interactions between parameters (i.e. t×y, t×s, y×s, t×y×s) (See Table 1) explained ASR (φ) by using program MARK [45]. As there was no site effect on ASR (ΔQAIC_c_ = 13.3), we pooled all individuals for the analysis. Using time-since-marking models, we obtained survival estimates for both recruits and returning males by segregating the bias occurring due to presence of recruits that were detected only once in our plots. It is impossible to build an estimable model incorporating the effects of age, time, and cohort, because those variables are a linear function of each other, we thus preferred to focus on age and time (Year) in this present study. Year-specific ASR was estimated for all intervals between breeding seasons (e.g. survival rate of individuals between 2006 and 2007). We determined the resighting probability (p) using a two-stage model selection process [1] by fitting the best parameterization of p and then fitting 22 candidate reduced models from that best global model. Because we spent approximately the same number of person-hours in all territories and that all territorial individuals were resighted at multiple occasions per breeding season (Table 1), we do not expect a difference in the probability of resighting recruits vs returning individuals. Consequently, we only compared the relative importance of treatment (t), year (y), the additive effect of both factors (t+y) and a null model (.) on resighting probability. The goodness of fit of the global model (i.e. φ_t*y*s_,p_y+t_) was tested using the median c-hat (*ĉ*) procedure [14]. The relative importance of each model was compared using the quasi-Akaike's Information Criterion adjusted for sample size (QAIC_c_). Models were considered to be equivalent when ΔQAIC_c_<2.0. Along with QAIC_c_, we used QAIC_c_ weights (Σ*w_i_*) to compare the support of each parameter. QAIC_c_ weights summed to 1 across the model set. QAIC_c_ weight for a given parameter was the sum of the QAIC_c_ weights of all models that included the parameter. The response variable with the largest predictor weight was considered to be the most important. Parameter estimates, standard deviation, and the 95% confidence intervals were generated for each variable of the best-ranked models. We also used an analysis of deviance (Anodev) implemented in program MARK to determine whether the temporal covariates (i.e. nest success and ENSO) had a significant effect on ASR [46]. This test compares the deviance explained by the covariate to unexplained deviance. The covariate would have been considered to have a significant effect on ASR if the associated p-value was lower than 0.05.

**Table 1:** Model parameters fitted in Cormack-Jolly-Seber models to assess their influence on apparent survival rate (ASR) of male Ovenbirds monitored from 2006–2014.

Parameter	Code	Prediction on ASR
Treatment	t	ASR will be lower in selection cut plots
Year	y	ASR will vary over the years
Status	s	ASR will be lower for recruits than for returning individuals
Daily nest survival rate	dns	ASR will be lower in the years following a season of low daily nest survival rate/nesting success rate
Nesting success	ns	
ENSO	e	ASR will be lower during La Niña phases
Treatment×year	t×y	ASR will be lower in selection cut plots during the first years post-harvest
Treatment×status	t×s	ASR will be lower for recruits in selection cut plots
Year×status	y×s	ASR will be lower for recruits depending on years

We also estimated the lifespan of individuals using the following equation:
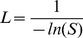
(1)where L is the estimated lifespan, and S is the estimated ASR.

Multi-state models would be inappropriate to our analysis because most variables have year- or site-specific values, except for the age at first capture, and parameter identifiability issues due to a high number of parameters would probably have occurred given the nature of our data. Robust design models also are not applicable in this case because we did not consider all the detections of an individual for a given breeding season as single capture events, and because we used both opportunistic and systematic resighting occasions to retrieve individual bands. Thus, we did not have proper, well-defined secondary resighting events, and the data did not fit the assumptions for the use of robust design models [47].

## Results

From 2006 through 2012, we captured and banded 177 male Ovenbirds in control plots and 153 in treated plots. Of the 98 Ovenbird males banded in the pre-harvest year (2006), 10 of 51 returned to control plots in 2012 and only 3 of 47 in treated plots, indicating a significant negative effect of selection harvesting (G-test = 5.15, *p* = 0.02). In 2014, only one of those 13 males returned to a control plot. Return rates do not account for imperfect detection, but this observation is interesting because it is a rare field test of the maximum longevity (11 years) known in this species [34]. These data indicate that individuals have reached at least 10 years of age. Apparent survival data were slightly over-dispersed (*ĉ* = 1.07), hence a correction for the lack of fit was required for our Cormack-Jolly-Seber models. Resighting probability was best explained by the null model and was high and relatively constant over time (model-averaged estimate: p = 0.76±0.02 SD).

**Figure 1:**
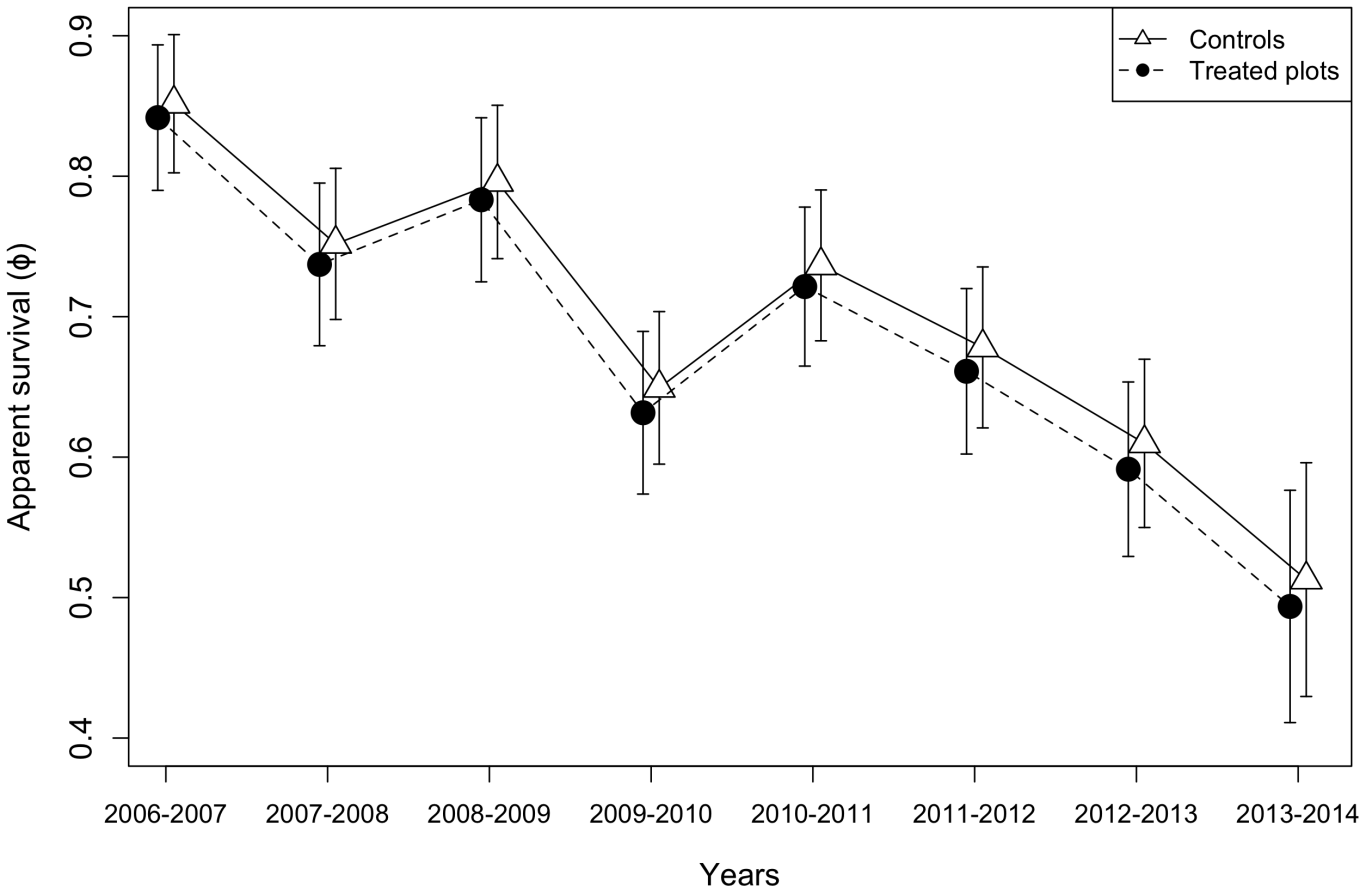
Temporal variation in apparent annual survival rates of Ovenbird males from control and treated plots, based on the second best model (φ_t+y_ p.). The treatment (selection harvesting; 30–40% basal area removal) was applied during the winter of 2006–2007.

The best-ranked model explaining variation in ASR was the year-dependent model, while the second best-ranked model included the additive effects of treatment and year (See Table 2). Year was by far the best predictor of ASR (Σ*w_i_* = 0.996). ASR was significantly higher during the first 3 years post-harvest than during subsequent years, as confidence intervals of those yearly parameters did not overlap zero (Figure 1, See Table S1). ASR declined from 0.85 to 0.50 (model φ_y_,p.) between 2006 and 2014, a 41% decline.

**Table 2:** Evaluation of mark-resighting models for male Ovenbirds monitored from 2006 to 2014 to assess variation in apparent annual survival (φ) and resighting probabilities (p).

Models	QAIC_c_	ΔQAIC_c_	*w_i_*	Parameters	Model deviance
**φ_y,_p_._**	**1418.21**	**0**	**0.577**	**9**	**411.88**
φ_y+T,_p.	1420.03	1.82	0.233	10	411.64
φ_s×t+y_,p.	1422.12	3.91	0.082	11	411.69
φ_s+y_,p.	1422.31	4.09	0.074	11	411.87
φ_s+T+y_,p.	1424.12	5.91	0.030	12	411.62
φ_._,p.	1430.59	12.38	0.001	2	438.48
φ_NS_,p.	1431.66	13.45	0.001	3	437.53
φ_dns_,p.	1432.11	13.90	0.001	3	437.97
φ_t_,p.	1432.17	13.96	0.001	3	438.04
φ_EnsO_,p.	1432.25	14.04	0.001	3	438.12
φ_S_,p.	1433.23	15.02	0.000	4	437.08
φ_s×t_,p.	1433.82	15.61	0.000	4	437.66
φ_s+t_,p.	1434.80	16.59	0.000	5	436.62
φ_t×y_,p.	1437.61	19.40	0.000	9	431.28
φ_s+t×y_,p.	1440.50	22.29	0.000	11	430.06
φ_s×t×y_,p.	1446.65	28.44	0.000	15	427.94
φ_s×y_,p.	1447.11	28.90	0.000	15	428.39
φ_T+s×y,_p.	1448.87	30.66	0.000	16	428.07
φ_s*t*y,_p.	1471.92	53.70	0.000	47	383.79
φ_s*t*y_,p_T_	1472.63	54.42	0.000	48	382.24
φ_s*t*y_,p_y_	1479.61	61.40	0.000	54	375.51
φ_s*t*y,_p_t+y_	1480.74	62.53	0.000	55	374.33

Symbols: φ = survival, p = resighting probability, (.) parameter constant, + = additive effect between two variables (e.g. T+Y), × = interaction effect between two variables (e.g. T×Y), * = full interaction between two parameters (e.g. T*Y = T+Y+T×Y).

Models were tested as functions of status, treatment, and annual variations (ENSO, daily nest survival, and nesting success). Bold type indicates the best-fit model. See Table 1 for meaning of codes.

On the basis of its QAIC_c_ weight, treatment was the second most influential variable to explain ASR (Σ*w_i_* = 0.263; See Table 2), and it was the only other variable included in the best-ranked models. As predicted, it was lower in treated plots than in controls (model φ_t_,p.: φ_treated_ = 0.69±0.03 SE; φ_control_ = 0.71±0.02). The estimated lifespan of individuals based on estimated ASR was 2.7 years in treated plots and 2.9 years in controls. However, the confidence interval around the parameter estimate β for treatment overlapped zero (−0.41; 0.20), indicating that the harvest treatment did not have a biologically significant effect even though ASR was consistently lower in treated plots by ca. 8%.

Male status received some support (Σ*w_i_* = 0.105). However, confidence intervals around those parameters included zero and male status was not included in any of the top two models. Models testing for effects of ENSO, nest survival, and nesting success on ASR had a very low QAIC_c_ and did not perform better than the null model. Furthermore, nest success (F_1,6_ = 0.22, p = 0.65), daily nest survival rate (F_1,6_ = 0.12, p = 0.75), and ENSO (F_1,6_ = 0.08, p = 0.78) had no significant effect on ASR (Table 2).

## Discussion

None of the hypothesized mechanisms explained observed variations in Ovenbird apparent survival rate (ASR) over the 9 years of this study. In fact, we observed a fairly continuous decline: at the end of the study period, the estimated ASR was only 41% of the initial value (Figure 1). Indeed, year was the best variable (Σ_wi_ = 0.996) to explain ASR. The trend in ASR could not be explained by (1) annual fluctuations in daily nest survival, (2) a treatment×year interaction that could have reflected shifts in the abundance of Ovenbird's main food (litter invertebrates), (3) the ENSO climate pattern, nor by (4) male status (returning male versus recruit).

If daily nest survival had driven changes in ASR, ASR values should have dropped following the summers of 2006, 2008, and especially 2012. Those were the years with the lowest daily nest survival (Table S2), reflecting peaks in abundance of important nest predators (e.g. eastern chipmunk, *Tamias striatus*; [48], A. Vernouillet & M.-A.Villard, unpubl. data). This was not the case. Indeed, models including annual variation in daily nest survival rate and nesting success received no support. In Pennsylvania, Bernard *et al.* [49] also reported no effect of an individual male Ovenbird's nesting success in the previous year on its probability of return.

There was also no evidence that selection harvesting influenced ASR. We hypothesized that it would influence ASR through a temporary reduction in food abundance in treated plots, and corresponding physiological costs associated with territory expansion. In the same study area, selection harvesting had a negative effect on Brown Creeper (*Certhia americana*) nest density [50] and nest provisioning rate [51], yet no effect on brood size at fledging (E. D'Astous and M.-A. Villard, unpublished data). Nonetheless, adult creepers from treated plots, as well as Ovenbirds, would be expected to spend more energy to feed their nestlings owing to greater distances travelled while foraging. In Ontario, Leshyk *et al.* [52] reported a higher stress level, based on corticosterone levels, in adult Ovenbirds captured in intensively-managed forest compared to birds from plots under lower-intensity management or control plots. Similarly, in the Eurasian Treecreeper (*C. familiaris*), nestlings born in small forest patches showed higher stress levels, according to the heterophil-lymphocytes ratio, than those from larger forest patches [53]. Males from treated plots had a slightly shorter lifespan (2.7 years vs 2.9 years) but it seems unlikely that they experienced a lower lifetime reproductive success given that the harvest treatment had no effect on per capita productivity [39].

Food abundance itself is known to influence avian breeding success [54]. Considering that Ovenbird males feed nestlings and fledglings [55], we predicted that they would suffer greater costs during years of low food abundance and, thus, return rates would be lower the following years. The abundance of litter invertebrates decreased significantly in the first year post-harvest in treated plots relative to controls, then increased gradually toward the level found in controls [39]. Yet, this gradual increase in food abundance did not seem to improve survival rate, which pursued its largely negative trend. In another study, experimental reduction in the abundance of foliage invertebrates did not result in lower ASR in the red-eyed vireo (*Vireo olivaceus*) [56].

Both recruits and returning individuals had a similar ASR (see Table 2, Σ_wi_ = 0.105). While some studies suggest that inexperienced individuals have a lower reproductive success [57]–[58] and, thus, may be more likely to emigrate [59], that was not the case in our study area. Our results suggest that breeding-site fidelity in Ovenbirds could develop over a single breeding season (see also [17]).

### El Niño Southern Oscillation

ASR appeared to covary in treated and control plots (Figure 1), a pattern suggestive of factors operating outside our study area. However, models including ENSO received no support. Using average SOI values from October to April, when Ovenbirds are presumed to be on their wintering grounds, the ENSO effect on ASR was also not significant (A. Vernouillet, S. Haché and M.-A. Villard, unpublished data). Similarly, a long-term study on Purple Martins (*Progne subis*), a species wintering from northeastern South America to southern Brazil [60], did not detect any relationship between ENSO and ASR [61]. In contrast, Sillett, Holmes, and Sherry [22] reported an important effect of ENSO on the ASR in the Black-throated Blue Warbler wintering in Jamaica, whereas the ASR of individuals on New Hampshire breeding grounds was relatively constant.

As insects are generally less sensitive to climatic fluctuations than fruits, insectivorous birds may not be strongly affected by ENSO (but see [25]). In a study conducted in Central America, there was no evidence for an influence of ENSO on body condition of insectivorous species [62]. In contrast, in a New Hampshire study area, annual variations in the abundance of insectivorous long-distance migrants (including the Ovenbird) were correlated with those of lepidopteran larvae [63], which in turn have been shown to fluctuate with the ENSO pattern [21]. In our study area, data on litter invertebrates actually suggested an effect of the harvest treatment, and no apparent fluctuations in control plots [39]. Hence, there was no evidence for an effect of climate patterns on Ovenbirds' main food source.

### Apparent survival rates recorded in the Ovenbird

In spite of the declining trend we observed, the ASR values we recorded (0.701; model φ.,p.) were higher than those previously reported for this species elsewhere. For example, long-term data from MAPS stations in northeastern North America indicate an ASR of 0.57±0.02 [64]. In Saskatchewan, Bayne and Hobson [29] reported a higher ASR among males occupying contiguous forest (0.62±0.06) than forest fragmented by agriculture (0.49±0.06) or by forestry (0.56±0.06) over a 4-year period. In Missouri, the ASR of male Ovenbirds monitored over 4 years was also lower than the one reported here (0.62±0.21; [65]). In the first 4 years of this study [33], the ASR was similar to that calculated over 9 years. The higher ASR we estimated may reflect the relatively high resighting probability (p = 0.76±0.02) we obtained compared to other studies, perhaps because we searched for banded individuals within a ca. 100 m-wide band around each site.

Because we could not distinguish emigration from mortality, the ultimate fate of missing birds remains unknown. However, the high resighting probability recorded in this study compared to other studies on the same species [28], [65]–[66] minimizes this bias. Each year, we searched for colour bands on all territorial individuals present in our study plots. The fact that territories of returning individuals generally overlapped between years [33] suggests a high degree of site fidelity in this population (see also [49]).

### Other potential factors explaining variations in ASR

Factors acting during migration such as tropical storms [67] or collisions with human infrastructures [68]–[70] might account for the observed annual fluctuations in ASR, but such occurrences are very difficult to relate to specific breeding populations. Parasites, including mites, and virus outbreaks such as those of the West Nile virus (WNV), might also explain some variation in ASR. However, WNV did not explain yearly fluctuations of ASR in Purple Martin [54] nor in the South Hills Crossbill (*Loxia curvirostra* complex, [71]).

From a statistical perspective, Cormack-Jolly-Seber models assume that all individuals have the same recapture and survival probabilities [1]. Violation of those assumptions (i.e. heterogeneity in the population) can hide the effects of some variables affecting only part of the population, and thus bias estimates of ASR [72]–[73]. To assess the robustness of our modelling results, we varied the *ĉ* value from 1 to 2 and the year-dependent model remained the best. Thus, we are confident that heterogeneity did not affect our conclusions.

## Conclusions

**Figure 2:**
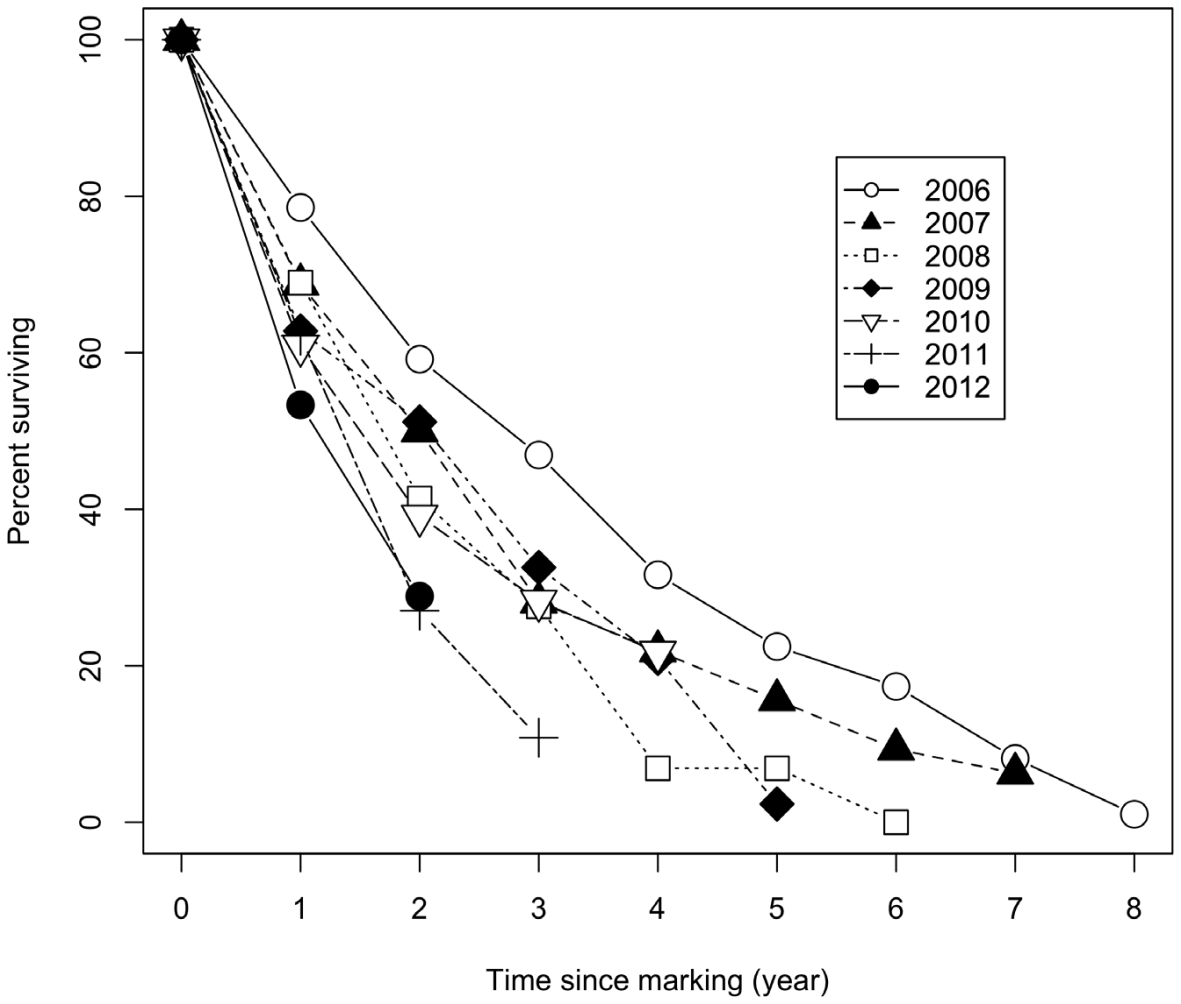
Survival curves of the seven cohorts of banded Ovenbird males pooled from the four pairs of plots, from capture (year 0) until 2014. Each curve corresponds to a separate cohort (i.e. group of newly-marked males during a breeding season, irrespective of their age) whose year of marking is indicated in the legend. Sample sizes per cohort were 98 (2006 cohort), 32 (2007), 29 (2008), 43 (2009), 46 (2010), 37 (2011), and 45 (2012).

We can only speculate about the factors underlying the nearly continuous declining trend we observed in the ASR of our focal Ovenbird population. This trend cannot be attributed to senescence because data were pooled across seven different cohorts and the oldest cohort (2006) did not decline at a faster rate (Figure 2). Thus, it did not unduly influence the overall trend. An avenue worth exploring is that of habitat changes at migratory stopover sites and in the main wintering area of our focal population. According to geolocator data retrieved from four individuals breeding in our study sites, the main wintering area of our study population would be Hispaniola (S. Haché, M.-A. Villard and E. Bayne, unpublished data). Hence, our results either suggest that wintering conditions have rapidly degraded in that region of the Caribbean, or along the migratory route used by these birds. Such changes would have to be much more dramatic than the ones underlying the long-term decline in Ovenbird capture rates reported in Puerto Rico [74], or the decline observed between 1976 and 2012 on the breeding grounds across Canada [75]. The high fidelity of adult birds for their wintering or even staging sites [76] may put breeding populations at risk when migratory connectivity is high. However, the stochasticity exhibited by juveniles in their selection of wintering sites [76] should provide some resilience against continued degradation of specific wintering sites. Such a buffering effect is suggested by the fact that recruitment rate tends to be fairly high in our study area following disturbance [33].

Until now, studies estimating ASR in migratory songbirds have mainly focused on events occurring on the breeding grounds (e.g. habitat disturbance, nest predation). Our results underline the importance of considering factors affecting ASR throughout the annual cycle, especially during migration and on the wintering grounds.

## Supporting Information

Table S1 Parameter estimates for best-ranked models explaining variation in ASR.
(DOCX)

Table S2 Annual covariates used to explain the variation in ASR.
(DOCX)

Table S3 Encounter history of Ovenbird males between 2006 to 2014.
(XLSX)
