# Correction: ENSO, Nest Predation Risk, Food Abundance, and Male Status Fail to Explain Annual Variations in the Apparent Survival Rate of a Migratory Songbird

**DOI:** 10.1371/journal.pone.0122941

**Published:** 2015-03-26

**Authors:** The PLOS ONE Staff

S1 Table, S2 Table, and S3 Table appear incorrectly. Please view the correct S1 Table, S2 Table, and S3 Table below.

## Supporting Information

S1 Table Parameter estimates (β_*i*_) for the two best-ranked models (ΔQAIC_c_ ≤ 2) explaining variation in apparent survival rate (ϕ) and resighting probabilities (p) of male Ovenbird from 2006–2014. Bold type indicates parameters that are biologically significant.
(DOCX)

S2 Table Annual covariates used to explain the variation in apparent survival rates (ASR) of Ovenbirds from 2006 to 2014. El Nino Southern Oscillation (ENSO) estimates are calculated by using the average Southern Oscillation Index (SOI) values from July to April of each year. Nesting success (NS) and daily nest survival rates (DNSR) were estimated using the logistic-exposure method.
(DOCX)

S3 Table Ovenbird individuals history between 2006 to 2014 in Black Brook District, New Brunswick. (NT—controls, T—treated plots; ASY—after-second year, SY—second year)
(XLSX)
