# Supplementary material for: ENSO, Nest Predation Risk, Food Abundance, and Male Status Fail to Explain Annual Variations in the Apparent Survival Rate of a Migratory Songbird
Source: PLoS One. 2014 Nov 24;9(11):e113844. doi: 10.1371/journal.pone.0113844 (PMC4242669; doi:10.1371/journal.pone.0113844)
Supplement: Table S1 — Parameter estimates for best-ranked models explaining variation in ASR. (DOCX) [file pone.0113844.s001.docx]

Table S1. Model parameters fitted in Cormack-Jolly-Seber models to assess their influence on apparent survival rate (ASR) of male Ovenbirds from 2006-2013.

| Parameter | Code | Prediction on ASR |
| --- | --- | --- |
| Treatment | t | ASR will be lower in selection cut plots |
| Year | y | ASR will vary over the years |
| Status | s | ASR will be lower for recruits than for returning individuals |
| Daily nest survival rate  Nesting success | dns  ns | ASR will be lower in the years following a season of low daily nest survival rate / nesting success rate |
| ENSO | e | ASR will be lower during La Niña phases |
| Treatment × year | t × y | ASR will be lower in selection cut plots during the first years post-harvest |
| Treatment × status | t × s | ASR will be lower for recruits in selection cut plots |
| Year × status | y × s | ASR will be lower for recruits depending on years |
