# Supplementary material for: ENSO, Nest Predation Risk, Food Abundance, and Male Status Fail to Explain Annual Variations in the Apparent Survival Rate of a Migratory Songbird
Source: PLoS One. 2014 Nov 24;9(11):e113844. doi: 10.1371/journal.pone.0113844 (PMC4242669; doi:10.1371/journal.pone.0113844)
Supplement: Table S2 — Annual covariates used to explain the variation in ASR. (DOCX) [file pone.0113844.s002.docx]

Table S2. Evaluation of mark-resighting models for male Ovenbirds from 2006 to 2013 used to assess variation in apparent annual survival (ϕ) and resighting probabilities (p). Models were tested as functions of status, treatment, and time. Bold type indicates the best-fit model. See Tab. 1 for meaning of codes.

| Models | QAIC_c_ | ΔQAIC_c_ | *w_i_* | Parameters | Model deviance |
| --- | --- | --- | --- | --- | --- |
| ϕ_t+y_p_._ | **1204.32** | **0** | **0.346** | **9** | **292.65** |
| ϕ_y_ p. | 1204.56 | 0.24 | 0.307 | 8 | 294.93 |
| ϕ_s×t+y_ p. | 1206.15 | 1.83 | 0.139 | 10 | 292.43 |
| ϕ_s+t+y_ p. | 1207.83 | 3.52 | 0.060 | 11 | 292.06 |
| ϕ_s+y_ p. | 1207.88 | 3.57 | 0.058 | 10 | 294.16 |
| ϕ_t_ p. | 1209.76 | 5.44 | 0.023 | 3 | 310.28 |
| ϕ. p. | 1210.21 | 5.89 | 0.018 | 2 | 312.74 |
| ϕ_dns_ p. | 1210.84 | 6.53 | 0.013 | 3 | 311.36 |
| ϕ_s×t_ p. | 1211.34 | 7.02 | 0.010 | 4 | 309.84 |
| ϕ_ns_ p. | 1212.16 | 7.84 | 0.007 | 3 | 312.67 |
| ϕ_e_ p. | 1212.22 | 7.90 | 0.007 | 3 | 312.74 |
| ϕ_s+t_ p. | 1212.58 | 8.26 | 0.006 | 5 | 309.05 |
| ϕ_s_ p. | 1212.84 | 8.52 | 0.005 | 4 | 311.33 |
| ϕ_t×y_ p. | 1214.45 | 10.13 | 0.002 | 8 | 304.82 |
| ϕ_s+t×y_ p. | 1217.88 | 13.56 | 0.000 | 10 | 304.16 |
| ϕ_s×t×y_ p. | 1223.72 | 19.40 | 0.000 | 13 | 303.83 |
| ϕ_t+s×y_ p. | 1224.00 | 19.68 | 0.000 | 14 | 302.03 |
| ϕ_s×y_ p. | 1224.11 | 19.79 | 0.000 | 13 | 304.21 |
| ϕ_s*t*y_ p_t_ | 1243.67 | 39.36 | 0.000 | 40 | 266.12 |
| ϕ_s*t*y_ p. | 1243.94 | 39.62 | 0.000 | 39 | 268.58 |
| ϕ_s*t*y_ p_y_ | 1249.59 | 45.27 | 0.000 | 45 | 260.93 |
| ϕ_s*t*y_ p _t+y_ | 1250.21 | 45.49 | 0.000 | 46 | 259.31 |

Parameter definitions: ϕ = survival, p = resighting probability, (.) parameter constant
