# Supplementary material for: ENSO, Nest Predation Risk, Food Abundance, and Male Status Fail to Explain Annual Variations in the Apparent Survival Rate of a Migratory Songbird
Source: PLoS One. 2014 Nov 24;9(11):e113844. doi: 10.1371/journal.pone.0113844 (PMC4242669; doi:10.1371/journal.pone.0113844)
Supplement: Table S3 — Encounter history of Ovenbird males between 2006 to 2014. (XLSX) [file pone.0113844.s003.xlsx]

Table S3. Parameter estimates (β*_i_*) for the three best-ranked models (ΔQAIC_c_ ≤ 2) explaining variation in apparent survival rate (ϕ) and resighting probabilities (p) of male Ovenbird from 2006-2013. Bold type indicates parameters that are biologically significant.

| Model | Label | β*_i_* | SE | 95% confidence limit | |
| --- | --- | --- | --- | --- | --- |
|  |  |  |  | lower | upper |
| ϕ_t+y_ p. | t | -0.260 | 0.172 | -0.596 | 0.077 |
|  | **y_1_** | **1.384** | **0.429** | **0.543** | **2.224** |
|  | **y_2_** | **0.762** | **0.369** | **0.039** | **1.485** |
|  | **y_3_** | **1.041** | **0.402** | **0.254** | **1.828** |
|  | y_4_ | 0.321 | 0.339 | -0.343 | 0.985 |
|  | y_5_ | 0.682 | 0.356 | -0.017 | 1.381 |
|  | y_6_ | 0.396 | 0.373 | -0.338 | 1.129 |
|  | y_7_ | 0.347 | 0.268 | -0.179 | 0.873 |
|  | **p** | **1.293** | **0.143** | **1.013** | **1.573** |
| ϕ_y_ p. | **y_1_** | **1.374** | **0.426** | **0.539** | **2.210** |
|  | **y_2_** | **0.785** | **0.367** | **0.065** | **1.506** |
|  | **y_3_** | **1.060** | **0.401** | **0.275** | **1.845** |
|  | y_4_ | 0.341 | 0.337 | -0.320 | 1.003 |
|  | y_5_ | 0.689 | 0.355 | -0.006 | 1.384 |
|  | y_6_ | 0.396 | 0.372 | -0.333 | 1.125 |
|  | y_7_ | 0.222 | 0.253 | -0.273 | 0.717 |
|  | **p** | **1.293** | **0.143** | **1.013** | **1.573** |
| ϕ_s×t+y_ p. | t×m | -0.095 | 0.276 | -0.635 | 0.445 |
|  | t×r | -0.390 | 0.410 | -1.194 | 0.414 |
|  | **y_1_** | **1.399** | **0.424** | **0.568** | **2.230** |
|  | **y_2_** | **0.805** | **0.368** | **0.083** | **1.526** |
|  | **y_3_** | **1.083** | **0.399** | **0.301** | **1.865** |
|  | y_4_ | 0.394 | 0.339 | -0.271 | 1.059 |
|  | **y_5_** | **0.719** | **0.354** | **0.024** | **1.413** |
|  | y_6_ | 0.445 | 0.373 | -0.87 | 1.176 |
|  | y_7_ | 0.246 | 0.258 | -0.258 | 0.751 |
|  | **p** | **1.308** | **0.143** | **1.027** | **1.589** |
