# Supplementary material for: Correction: ENSO, Nest Predation Risk, Food Abundance, and Male Status Fail to Explain Annual Variations in the Apparent Survival Rate of a Migratory Songbird
Source: PLoS One. 2015 Mar 26;10(3):e0122941. doi: 10.1371/journal.pone.0122941 (PMC4374869; doi:10.1371/journal.pone.0122941)
Supplement: S1 Table — Bold type indicates parameters that are biologically significant. (DOCX) [file pone.0122941.s001.docx]

Table S1. Parameter estimates (β*_i_*) for the two best-ranked models (ΔQAIC_c_ ≤ 2) explaining variation in apparent survival rate (ϕ) and resighting probabilities (p) of male Ovenbird from 2006-2014. Bold type indicates parameters that are biologically significant.

| Model | Label | β*_i_* | SE | 95% confidence limit | |
| --- | --- | --- | --- | --- | --- |
|  |  |  |  | Lower | upper |
| ϕ_y_ p. | **_Y1_** | **1.698** | **0.479** | **0.759** | **2.636** |
|  | **y_2_** | **1.068** | **0.413** | **0.259** | **1.877** |
|  | **y_3_** | **1.320** | **0.444** | **0.449** | **2.191** |
|  | y_4_ | 0.574 | 0.381 | -0.174 | 1.321 |
|  | **y_5_** | **0.983** | **0.403** | **0.194** | **1.772** |
|  | y_6_ | 0.696 | 0.392 | -0.073 | 1.466 |
|  | y_7_ | 0.401 | 0.418 | -0.419 | 1.222 |
|  | y_8_ | 0.011 | 0.310 | -0.597 | 0.619 |
|  | **p** | **1.171** | **0.126** | **0.925** | **1.417** |
| ϕ_y+T_ p. | _T_ | -0.077 | 0.158 | -0.387 | 0.233 |
|  | **y_1_** | **1.696** | **0.480** | **0.756** | **2.636** |
|  | **y_2_** | **1.057** | **0.414** | **0.245** | **1.868** |
|  | **y_3_** | **1.310** | **0.445** | **0.437** | **2.182** |
|  | y_4_ | 0.564 | 0.383 | -0.185 | 1.314 |
|  | **y_5_** | **0.977** | **0.404** | **0.185** | **1.768** |
|  | y_6_ | 0.694 | 0.393 | -0.077 | 1.464 |
|  | _Y7_ | 0.395 | 0.420 | -0.428 | 1.218 |
|  | _Y8_ | 0.051 | 0.322 | -0.581 | 0.683 |
|  | **p** | **1.170** | **0.126** | **0.924** | **1.416** |
