# Supplementary material for: Correction: ENSO, Nest Predation Risk, Food Abundance, and Male Status Fail to Explain Annual Variations in the Apparent Survival Rate of a Migratory Songbird
Source: PLoS One. 2015 Mar 26;10(3):e0122941. doi: 10.1371/journal.pone.0122941 (PMC4374869; doi:10.1371/journal.pone.0122941)
Supplement: S2 Table — El Nino Southern Oscillation (ENSO) estimates are calculated by using the average Southern Oscillation Index (SOI) values from July to April of each year. Nesting success (NS) and daily nest survival rates (DNSR) were estimated using the logistic-exposure method. (DOCX) [file pone.0122941.s002.docx]

Table S2. Annual covariates used to explain the variation in apparent survival rates (ASR) of Ovenbirds from 2006 to 2014.El Nino Southern Oscillation (ENSO) estimates are calculated by using the average Southern Oscillation Index (SOI) values from July to April of each year. Nesting success (NS) and daily nest survival rates (DNSR) were estimated using the logistic-exposure method.

| Year | ENSO | NS | DNSR |
| --- | --- | --- | --- |
| 2006-2007 | -0.75 | 0.2122 | 0.9442 |
| 2007-2008 | 1.66 | 0.5034 | 0.9749 |
| 2008-2009 | 1.78 | 0.2004 | 0.9422 |
| 2009-2010 | -0.69 | 0.3027 | 0.9567 |
| 2010-2011 | 3.51 | 0.5424 | 0.9776 |
| 2011-2012 | 1.35 | 0.4697 | 0.9724 |
| 2012-2013 | 0.23 | 0.0592 | 0.9006 |
| 2013-2014 | 0.56 | 0.7338 | 0.9886 |
